# A prognosis-related molecular subtype for early-stage non-small lung cell carcinoma by multi-omics integration analysis

**DOI:** 10.1186/s12885-021-07846-0

**Published:** 2021-02-06

**Authors:** Kai Yang, Ying Wu

**Affiliations:** 1 Shenzhen Institute of Respiratory Diseases, Shenzhen People’s Hospital (the Second Clinical Medical College, Jinan University; the First Affiliated Hospital, Southern University of Science and Technology), Shenzhen, 518055 China; 2 Department of Biostatistics, School of Public Health, Southern Medical University, Guangzhou, 510080 China

**Keywords:** Early-stage lung cancer, Molecular subtype, Prognosis, Gene expression, DNA methylation

## Abstract

**Background:**

Early-stage non-small cell lung carcinoma (NSCLC) accounts for more than 80% of lung cancer, which is a kind of cancer with high heterogeneity, so the genetic heterogeneity and molecular subtype should be explored.

**Methods:**

Partitioning Around Medoid algorithm was used to acquire the molecular subtype for early-stage NSCLC based on prognosis-related mRNAs and methylation sites. Random forest (RF) and support vector machine (SVM) were used to build prediction models for subtypes.

**Results:**

Six prognosis-related subtypes for early-stage NSCLC, including 4 subtypes for lung squamous cell carcinoma (LUSC) and 2 subtypes for lung adenocarcinoma (LUAD), were identified. There were highly expressed and hypermethylated gene regions for LUSC-C1 and LUAD-C2, highly expressed region for LUAD-C1, and hypermethylated regions for LUSC-C3 and LUSC-C4. Molecular subtypes for LUSC were mainly determined by DNA methylation (14 mRNAs and 362 methylation sites). Molecular subtypes for LUAD were determined by both mRNA and DNA methylation information (143 mRNAs and 458 methylation sites). Ten methylation sites were selected as biomarkers for prediction of LUSC-C1 and LUSC-C3, respectively. Nine genes and 1 methylation site were selected as biomarkers for LUAD subtype prediction. These subtypes can be predicted by the selected biomarkers with RF and SVM models.

**Conclusions:**

In conclusion, we proposed a prognosis-related molecular subtype for early-stage NSCLC, which can provide important information for personalized therapy of patients.

**Supplementary Information:**

The online version contains supplementary material available at 10.1186/s12885-021-07846-0.

## Background

Non-small cell lung carcinoma (NSCLC) accounts for more than 80% of lung cancer, which is the second most common cancer and the most common cause of cancer-associated deaths worldwide [1, 2]. With the development of diagnostic techniques, more NSCLC patients will be diagnosed at earlier stage [3, 4]. These patients can achieve a relatively superior prognosis, but some patients still develop recurrent cancer and about 40% of them will die of cancer within 5 years [5, 6]. NSCLC is also a kind of cancer with high heterogeneity, of which 45% were lung squamous cell carcinoma (LUSC) and 30% were lung adenocarcinoma (LUAD) [7]. Histological and genetic diversity can account for some of the individual variation in NSCLC survival. Therefore, identification of molecular subtype for early-stage NSCLC patients associated with survival will benefit early treatment and patient prognosis.

Molecular subtype has been used in the exploration of NSCLC heterogeneity. Gene expression subtypes of LUSC and LUAD have been proposed by The Cancer Genome Atlas (TCGA) research network, respectively [8, 9]. A multiplatform-based NSCLC molecular subtype including 9 subtypes for 1023 NSCLC patients has also been identified in a recent study [10]. There are some other kinds of lung cancer molecular subtypes according to different gene sets [11, 12]. However, there were some special molecular characteristics for early-stage NSCLC. Patient prognostic information has also not well utilized in these subtypes and gene sets, leading to weak predictive ability for patient prognosis.

In this study, we analyzed gene expression and DNA methylation data for early-stage NSCLC, and proposed a prognosis-related molecular subtypes for LUSC and LUAD. Then, we explored the function of differentially expressed genes and differentially methylated genes. We also selected biomarkers and built prediction model for each subtype in training dataset, and validated the models in test dataset. The prediction model was evaluated by sensitivity (SE), specificity (SP) and area under the ROC curve (AUC). Furthermore, we analyzed the molecular functions of these biomarkers in cancer development.

## Methods

### Datasets and preprocessing

RNA-Seq data, DNA methylation data and clinical information of NSCLC patients were downloaded from the UCSC Xena website (http://xena.ucsc.edu/). The RNA-seq data were log_2_ transformed RSEM normalized counts and mapped to HUGO gene symbols. The DNA methylation levels were represented by β-values (from 0 to 1). Methylation sites were filtered by the following criteria: 1) probes located in the X or Y chromosome; 2) SNP present within the assay of probe; 3) probes did not annotate with any reference genes; 4) probes located in the shelves and oversea regions of CpG island. Genes and methylation sites with missing value in more than 20% of patients were excluded, and patients without mRNA data or methylation data were also removed from further analysis. Data were centralized and standardized before analysis.

### Molecular subtypes related with overall survival

For each gene and methylation site in the entire data set, we built a univariate Cox proportional hazard (Cox-PH) model and selected variables with *P* values less than 0.001. We than used these genes and methylation sites to cluster the patients using Partitioning Around Medoid (PAM) clustering algorithm. The cluster number K of PAM clustering algorithm was set to 2–5. The optimal number of NSCLC clusters was determined by maximizing the difference of overall survival among different subtypes. The Database for Annotation, Visualization and Integrated Discovery (version 6.8, DAVID) tool was used for the functional annotation for Gene Ontology (GO) terms and Kyoto Encyclopedia of Genes and Genomes (KEGG) pathways.

### Prediction model for molecular subtypes

We randomly divided the data set into training set and test set, in which training set contained 60% patients (Table 1). We selected biomarkers and built prediction models for molecular subtypes in training set, and validated the models in test set. In the biomarker selection phase, univariate Wilcoxon test was firstly used to selected differentially expressed genes and methylated sites (*P* < 0.001) compared with other subtypes in the training dataset. Then, a multivariate partial least square (PLS) model was established, and 10 variables with largest variable important projection (VIP) values were selected as biomarkers for each subtype. Random forest (RF) and support vector machine (SVM) models were constructed with 10 selected biomarkers in training dataset. The model prediction ability was evaluated in training and test datasets, respectively.

**Table 1:** Clinical characteristics of early-stage NSCLC patients in training and test sets

		LUSC		LUAD	
		Training set	Test set	Training set	Test set
N		181	122	210	141
Age		68.41 ± 8.41	66.93 ± 9.48	65.43 ± 9.66	65.46 ± 9.98
Sex	Female	48 (26.52)	32 (26.23)	117 (55.71)	73 (51.77)
	Male	133 (73.48)	90 (73.77)	93 (44.29)	68 (48.23)
Pathologic stage	I	101 (55.80)	69 (56.56)	140 (66.67)	99 (70.21)
	II	80 (44.20)	53 (43.44)	70 (33.33)	42 (29.79)
Therapy outcome^a^	CR	128 (88.28)	89 (85.58)	138 (77.97)	99 (81.15)
	PR	1 (0.69)	3 (2.88)	1 (0.56)	2 (1.64)
	SD	5 (3.45)	7 (6.73)	17 (9.60)	4 (3.28)
	PD	11 (7.59)	5 (4.81)	21 (11.86)	17 (13.93)
Smoking status^b^	1	5 (2.84)	5 (4.20)	33 (16.26)	17 (12.32)
	2	62 (35.23)	35 (29.41)	48 (23.65)	37 (26.81)
	3	30 (17.05)	18 (15.13)	54 (26.60)	37 (26.81)
	4	77 (43.75)	60 (50.42)	66 (32.51)	45 (32.61)
	5	2 (1.14)	1 (0.84)	2 (0.99)	2 (1.45)
Pack year		52.20 ± 27.93	52.65 ± 28.92	39.18 ± 24.70	43.81 ± 28.38

The clinical characteristics were not statistically significant between training and test sets (*P* > 0.05). ^a^ *CR* complete response, *PR* partial response, *SD* stable disease, *PD* progressive disease. ^b^ 1: Lifelong non-smoker; 2: Current smoker; 3: Current reformed smoker for > 15 years; 4: Current reformed smoker for ≤15 years; 5: Current reformed smoker, duration not specified

### Evaluation criteria

The prediction model performance was evaluated by sensitivity (SE), specificity (SP) and area under the ROC curve (AUC). SE and SP were defined by:

$$ SE=\frac{TP}{TP+ FN} $$

$$ SP=\frac{TN}{TN+ FP} $$

where.

True Positive (TP): the patient belongs to a subtype, and the prediction model predicts the patient as this subtype;

False Positive (FP): the patient does not belong to a subtype, but the prediction model predicts the patient as this subtype;

True Negative (TN): the patient does not belong to a subtype, and the prediction model does not predict the patient as this subtype;

False Negative (FN): the patient belongs to a subtype, but the prediction model does not predict the patient as this subtype.

AUC were defined by:

$$ AUC=\frac{\sum {r}_i-{n}_0\left({n}_0+1\right)/2}{n_0{n}_1} $$

where *n*_0_ and *n*_1_ are the number of patients who belong to and not belong to a subtype respectively, and *r*_*i*_ is the rank of *i*_*th*_ patient of a subtype in the ranked list.

## Results

### Prognosis-related molecular subtypes

**Fig. 1:**
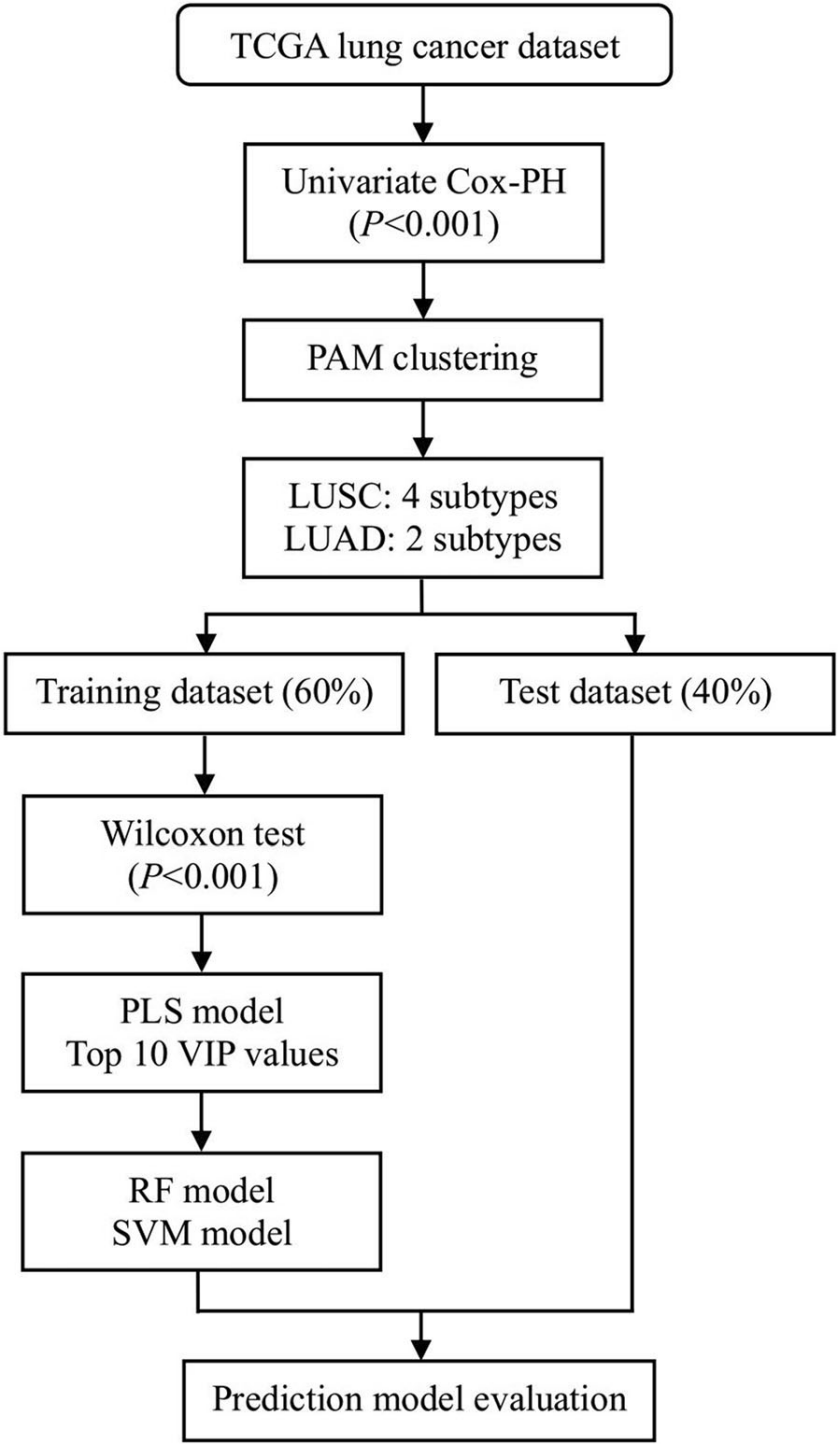
An overview workflow of prognosis-related molecular subtypes identification and biomarkers selection in early-stage NSCLC

An overview workflow of this study was shown in Fig. 1. In total, 303 LUSC and 351 LUAD patients with coupled mRNA and methylation data were analyzed in this study. After screening according to the criteria in the “method” section, 16249 mRNAs and 162,926 methylation sites were included. In the univariate Cox regression analysis, 14 mRNAs and 362 methylation sites in LUSC, and 143 mRNAs and 458 methylation sites in LUAD were associated with overall survival at the 0.001 level.

**Fig. 2:**
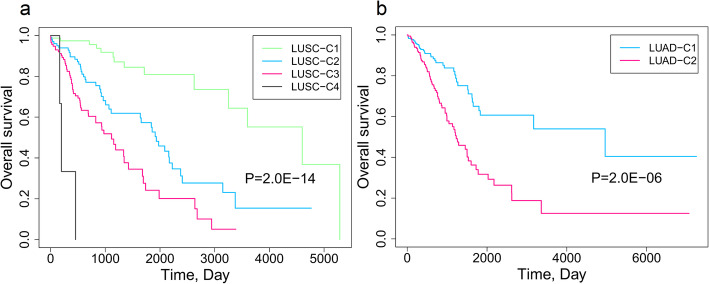
The association between molecular subtypes and overall survival. **a** LUSC. **b** LUAD

**Fig. 3:**
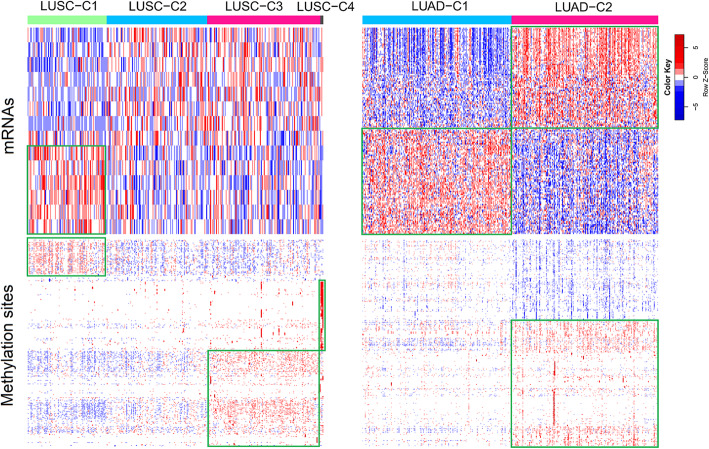
The heatmap plot of mRNAs and methylation sites in different subtypes of NSCLC. Highly expressed and hypermethylated gene regions were marked with green boxes

In the identification of prognosis-related molecular subtypes, the genes and methylation sites related with overall survival were used in the PAM clustering analysis, with cluster number K ranging from 2 to 5 (Supplementary Fig. 1). In the Kaplan-Meier (K-M) survival curves of LUSC and LUAD patients for different cluster number K, optimum survival curves were identified by 4 clusters for LUSC and 2 clusters for LUAD, respectively (Fig. 2). These subtypes showed different gene expression and DNA methylation patterns (Fig. 3). There were highly expressed and hypermethylated gene regions for LUSC-C1 and LUAD-C2, highly expressed region for LUAD-C1, and hypermethylated regions for LUSC-C3 and LUSC-C4.

### Functional annotation of differentially expressed genes and methylated genes related with overall survival

Functional enrichment was conducted for genes and methylated genes related with overall survival in LUSC and LUAD. Firstly, 362 and 458 methylation sites in LUSC and LUAD were located in 267 and 339 genes, respectively. The distribution of methylation sites was showed in Supplementary Fig. 2. The methylated genes in LUSC were significantly enriched in 8 GO biological process terms, 4 GO cellular component terms, and 7 GO molecular function terms (Supplementary Table 1). The 14 differentially expressed genes were also enriched in 3 GO cellular component terms at 0.2 level.

For LUAD patients, the methylated genes were significantly enriched in 11 biological process terms, 9 cellular component terms, 7 molecular function terms, and 2 KEGG pathways (Supplementary Table 2). The differentially expressed genes were significantly enriched in 21 biological process terms, 12 cellular component terms, 11 molecular function terms, and 7 KEGG pathways.

### Biomarkers for the prediction of subtypes

We next sought to select specific biomarkers and build prediction models for molecular subtypes. There were only 4 patients and obvious hypermethylated region in LUSC-C4 subtype, so we selected biomarkers and built prediction model for LUSC-C1, LUSC-C3 and LUAD. In the training dataset which included 60% patients, univariate Wilcoxon test was firstly used, and 114, 182, 303 biomarkers with *P* < 0.001 were selected for LUSC-C1, LUSC-C3 and LUAD. Then, these biomarkers were further used to build a multivariate PLS model and 10 variables with VIP values were selected as biomarkers for each subtype, respectively (Table 2). Ten methylation sites were selected as biomarkers for prediction of LUSC-C1 and LUSC-C3, respectively. Seven biomarkers (cg00894870, cg03041700, cg04738309, cg08356572, cg09844983, cg11416447 and cg22627950) were down-regulated, and 3 biomarkers (cg15987088, cg22627950 and cg24599434) were up-regulated in LUSC-C1 (Supplementary Fig. 3). Two biomarkers (cg02074191 and cg12065562) were down-regulated, and 8 biomarkers (cg00431236, cg00894870, cg02590972, cg03041700, cg04417954, cg08356572, cg11416447 and cg22627950) were up-regulated in LUSC-C3 (Supplementary Fig. 4). Nine genes and 1 methylation site were selected as biomarkers for LUAD subtype prediction. These biomarkers were all down-regulated in LUAD-C1 (Supplementary Fig. 5).

**Table 2:** Biomarkers for prediction of LUAD and LUSC subtypes

	ID_REF	Chr.	Gene name	Genetic location	Epigenetic location
LUSC-C1	cg00894870	1	MRTO4; KIAA0090	Body; TSS1500	Island
	cg03041700	1	ATAD3B	Body	Island
	cg04738309	5	C5orf13	Body	Island
	cg08356572	6	TRIM27	1stExon	Island
	cg09844983	1	RPA2	Body	Island
	cg11416447	7	DMTF1	TSS200	N_Shore
	cg15987088	3	GHSR	TSS200	Island
	cg17152757	3	GHSR	TSS200	Island
	cg22627950	7	TMED4	TSS1500	Island
	cg24599434	3	GHSR	TSS200	Island
LUSC-C3	cg00431236	2	ACP1; SH3YL1	1stExon; TSS1500	Island
	cg00894870	1	MRTO4; KIAA0090	Body; TSS1500	Island
	cg02074191	5	PCDHGA1; PCDHGA2; PCDHGA3; PCDHGA4; PCDHGA5; PCDHGA6; PCDHGA7; PCDHGA8; PCDHGB1; PCDHGB2; PCDHGB3; PCDHGB4; PCDHGB5	Body; Body; Body; Body; Body; Body; Body; Body; Body; Body; Body; Body; 1stExon	Island
	cg02590972	2	RPL37A	TSS200	N_Shore
	cg03041700	1	ATAD3B	Body	Island
	cg04417954	15	CRTC3	Body	Island
	cg08356572	6	TRIM27	1stExon	Island
	cg11416447	7	DMTF1	TSS200	N_Shore
	cg12065562	5	PCDHB18	Body	Island
	cg22627950	7	TMED4	TSS1500	Island
LUAD	ANLN	7			
	CCNA2	4			
	CDCA5	11			
	CKAP2L	2			
	DLGAP5	14			
	KIF4A	X			
	KPNA2	17			
	SHCBP1	16			
	TPX2	20			
	cg20097219	17	TBC1D16	Body	S_Shore

To evaluate the predictive performance of biomarkers, RF and SVM models for subtype prediction were built, and SE, SP and AUC were calculated in training and test datasets (Table 3). As results, we got a good prediction performance in the both training and test datasets. AUCs for LUSC-C1, LUSC-C3 and LUAD in RF model were 0.84, 0.77 and 0.83, whereas 0.85, 0.85 and 0.83 in SVM model, respectively. The prediction model built by SVM model was superior to the one built by RF model. These subtypes were significantly associated with overall survival, except for LUAD subtypes in test set, which also tended to be significant (Supplementary Fig. 6).

**Table 3:** Diagnostic capacities of prediction model for molecular subtypes of LUSC and LUAD

	Comparison group	Prediction model	Training dataset			Test dataset		
			SE	SP	AUC (95% CI)^*^	SE	SP	AUC (95% CI)^*^
LUSC	C1 vs C2 and C3	RF	1	1	1	0.72	0.96	0.84 (0.76–0.92)
		SVM	0.93	0.90	0.93 (0.89–0.98)	0.75	0.96	0.85 (0.77–0.93)
	C3 vs C1 and C2	RF	1	1	1	0.69	0.86	0.77 (0.68–0.86)
		SVM	0.88	0.96	0.92 (0.87–0.97)	0.81	0.89	0.85 (0.78–0.93)
LUAD	C1 vs C2	RF	1	1	1	0.86	0.80	0.83 (0.76–0.89)
		SVM	0.90	0.89	0.90 (0.85–0.94)	0.81	0.85	0.83 (0.77–0.89)

^*^ *P* < 0.05. *CI* confidence interval, *RF* random forest, *SVM* support vector machine

## Discussion

In this study, we proposed a prognosis-related molecular subtype for early-stage NSCLC, including 4 subtypes for LUSC and 2 subtypes for LUAD. These subtypes showed different trend in overall survival, gene expression pattern, and DNA methylation level. Most subtypes showed highly expressed and hypermethylated gene regions, which facilitated the biomarker selection for subtypes. We also selected biomarkers and built prediction models with good performance, which can help the grouping of new patients and therapy strategy selection.

LUSC patients were divided into 4 clusters by 14 mRNAs and 362 methylation sites related with overall survival. These subtypes were mainly determined by DNA methylation information, and all the selected biomarkers were also methylation sites. Five methylation sites (cg00894870, cg03041700, cg08356572, cg11416447 and cg22627950) were selected as biomarkers for both LUSC-C1 and LUSC-C3, in which the function of 4 methylated genes were associated with cancer [13–18]. The function of 267 genes were mainly associated with regulation of cell cycle and gene transcription.

In LUSC-C1, 3 hyper-methylated sites were located in transcriptional start site (TSS) 200 regions of GHSR and weakly negatively related with GHSR (Supplementary Table 3), which can encode growth hormone secretagogue receptor (GHS-R) and related with energy metabolism. KIAA0090, ATAD3B, TRIM27 and DMTF1, regulated by hypo-methylated sites, were also associated with cancer. KIAA0090, which was positively related with hypo-methylated cg00894870, was associated with cancer metastasis and prognosis [16]. ATAD3B was expressed in cancer cell, and may related with tumorigenesis, proliferation and chemoresistance [14, 15]. TRIM27 was an oncogene [18] and DMTF1 can regulated ARF-p53 pathway [13, 17].

In LUSC-C3, 8 hyper-methylated sites were located in 10 genes. In addition to 4 same genes (KIAA0090, ATAD3B, TRIM27 and DMTF1) with LUSC-C1, ACP1 and SH3YL1 also played important roles in cancer. ACP1 can encode a tyrosine phosphatase, which was an anti-tumorigenic factor interacted with PDGF-R and FAK [19]. SH3YL1 can regulate migration of cancer cell [20]. Two hypo-methylated sites were located in gene body of PCDH gene family (PCDHA, PCDHB and PCDHG). The aberrant methylations of these genes were also found in breast cancer [21].

Unlike LUSC, LUAD patients were divided into 2 clusters by 143 mRNAs and 458 methylation sites, which indicated that these subtypes were determined by both mRNA and DNA methylation. These differentially expressed genes were mainly associated with cell cycle regulation. Whereas the differentially methylated genes were involved in a variety of GO terms and KEGG pathways, such as signal transduction, cell division and apoptosis.

In LUAD-C1, 10 selected biomarkers were all down-regulated in LUAD-C1. ANLN, CCNA2, CDCA5, DLGAP5, TPX2 and KIF4A were involved in the regulation of cell cycle (Supplementary Table 4). CKAP2L and SHCBP1 were associated with spindle formation, which was also involved in cell cycle. In previous study, over expression of 9 selected gene biomarkers (ANLN, CCNA2, CDCA5, CKAP2L, DLGAP5, KIF4A, KPNA2, SHCBP1 and TPX2) can indicate poor prognosis in different cancer types, including lung cancer, colon cancer, breast cancer and bladder cancer [22–31].

We built 2 prediction models for subtype prediction based on RF and SVM algorithms. The SE, SP and AUC for subtype prediction in training dataset were 1 by RF model, larger than the values calculated by SVM model. However, these values were smaller than those calculated by SVM model in test dataset. This phenomenon indicated that the model built by RF was over-fitting, and the prediction ability was worse for new data than SVM model.

## Conclusions

In conclusion, we identified 6 subtypes for early-stage NSCLC, including 4 subtypes for LUSC and 2 subtypes for LUAD, by gene expression and DNA methylation data integration analysis. Furthermore, we also selected biomarkers and built prediction model to distinguish these subtypes, and most of these biomarkers were involved in tumor related function.

## Supplementary Information

**Additional file 1: Supplementary Fig. 1**. Survival curves for different number of molecular subtypes. **Supplementary Fig. 2**. The distribution of methylation sites. **Supplementary Fig. 3**. Boxplots of 10 methylation site biomarkers for LUSC-C1. **Supplementary Fig. 4**. Boxplots of 10 methylation site biomarkers for LUSC-C3. **Supplementary Fig. 5**. Boxplots of 9 gene expression and 1 methylation site biomarkers for LUAD-C1. **Supplementary Fig. 6**.The K-M survival curve for molecular subtypes and overall survival in different data set. (A) LUSC in training set. (B) LUAD in training set. (C) LUSC in test set. (D) LUAD in test set. **Supplementary Table 1**. Functional enrichment of differentially expressed genes and methylated genes related with overall survival for LUSC. **Supplementary Table 2**. Functional enrichment of differentially expressed genes and methylated genes related with overall survival for LUAD. **Supplementary Table 3**. The correlation of methylation sites and located genes. **Supplementary Table 4**. Functional enrichment of 9 biomarkers for LUAD-C1.

## Data Availability

Public access to the databases of this study is open. The data that support the findings of this study are available at https://xenabrowser.net/datapages/?cohort=TCGA%20Lung%20Adenocarcinoma%20(LUAD) and https://xenabrowser.net/datapages/?cohort=TCGA%20Lung%20Squamous%20Cell%20Carcinoma%20(LUSC).

## Abbreviations

AUC: Area under the ROC curve
DAVID: The Database for Annotation, Visualization and Integrated Discovery
FN: False negative
FP: False positive
GO: Gene Ontology
KEGG: Kyoto Encyclopedia of Genes and Genomes
LUAD: Lung adenocarcinoma
LUSC: Lung squamous cell carcinoma
NSCLC: Non-small cell lung carcinoma
PLS: Partial least square
RF: Random forest
SE: Sensitivity
SP: Specificity
SVM: Support vector machine
TCGA: The Cancer Genome Atlas
TN: True negative
TP: True positive
VIP: Variable important projection
