# Supplementary material for: A prognosis-related molecular subtype for early-stage non-small lung cell carcinoma by multi-omics integration analysis
Source: BMC Cancer. 2021 Feb 6;21:128. doi: 10.1186/s12885-021-07846-0 (PMC7866742; doi:10.1186/s12885-021-07846-0)
Supplement: Supplementary file 1 — Additional file 1: Supplementary Fig. 1. Survival curves for different number of molecular subtypes. Supplementary Fig. 2. The distribution of methylation sites. Supplementary Fig. 3. Boxplots of 10 methylation site biomarkers for LUSC-C1. Supplementary Fig. 4. Boxplots of 10 methylation site biomarkers for LUSC-C3. Supplementary Fig. 5. Boxplots of 9 gene expression and 1 methylation site biomarkers for LUAD-C1. Supplementary Fig. 6.The K-M survival curve for molecular subtypes and overall survival in different data set. (A) LUSC in training set. (B) LUAD in training set. (C) LUSC in test set. (D) LUAD in test set. Supplementary Table 1. Functional enrichment of differentially expressed genes and methylated genes related with overall survival for LUSC. Supplementary Table 2. Functional enrichment of differentially expressed genes and methylated genes related with overall survival for LUAD. Supplementary Table 3. The correlation of methylation sites and located genes. Supplementary Table 4. Functional enrichment of 9 biomarkers for LUAD-C1. [file 12885_2021_7846_MOESM1_ESM.docx]

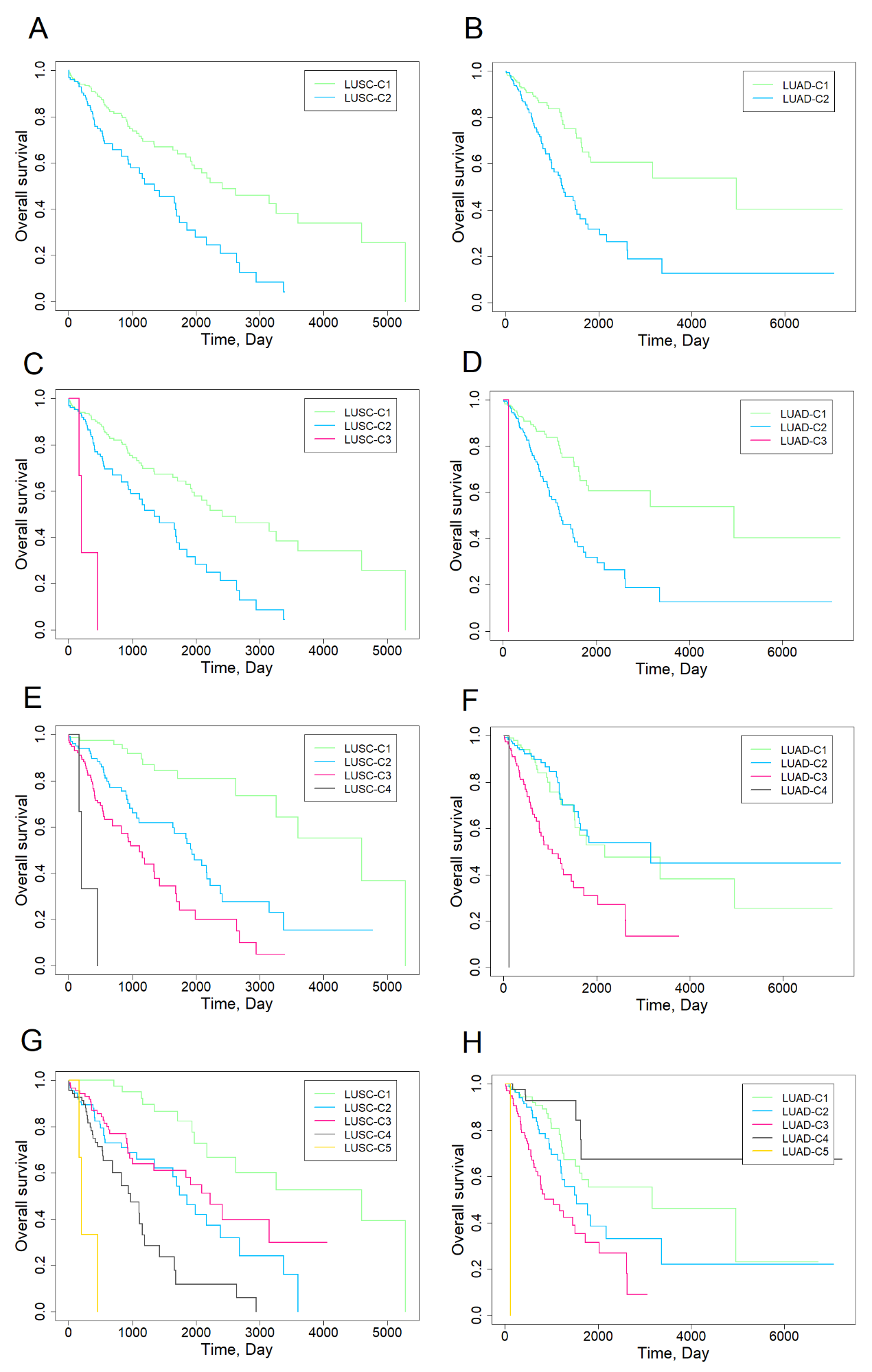


Supplementary fig. 1. Survival curves for different number of molecular subtypes.


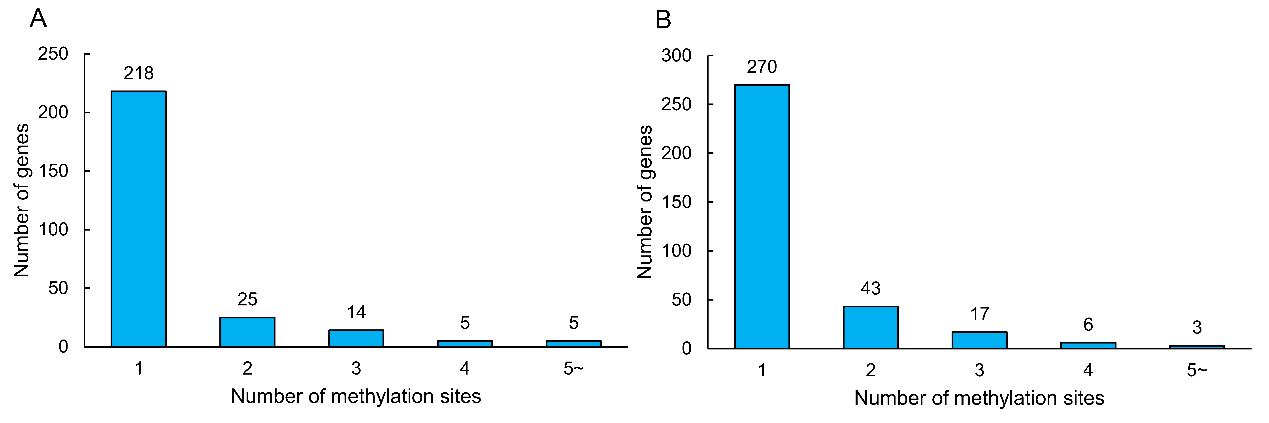


Supplementary fig. 2. The distribution of methylation sites.


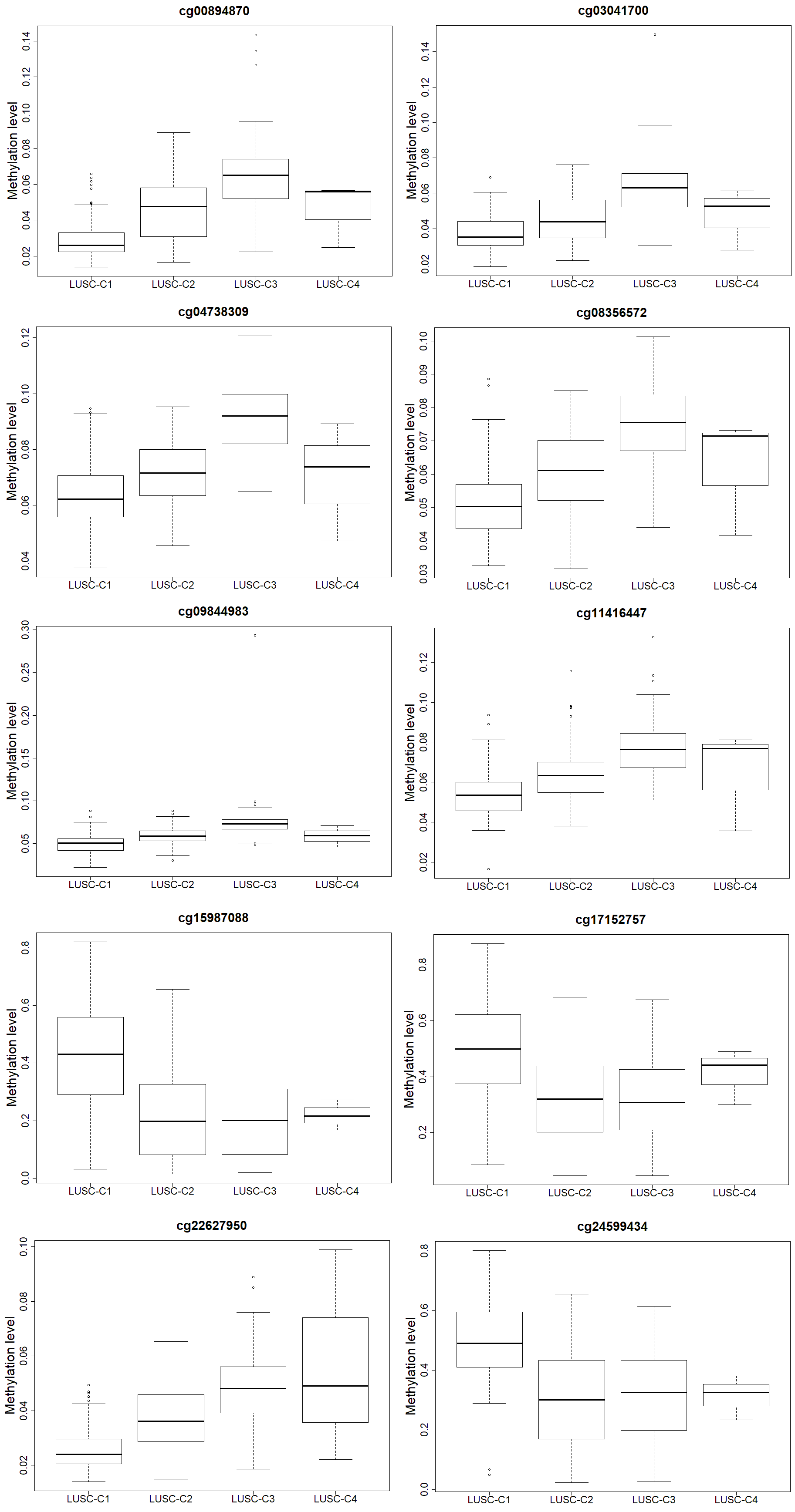


Supplementary fig. 3. Boxplots of 10 methylation site biomarkers for LUSC-C1.


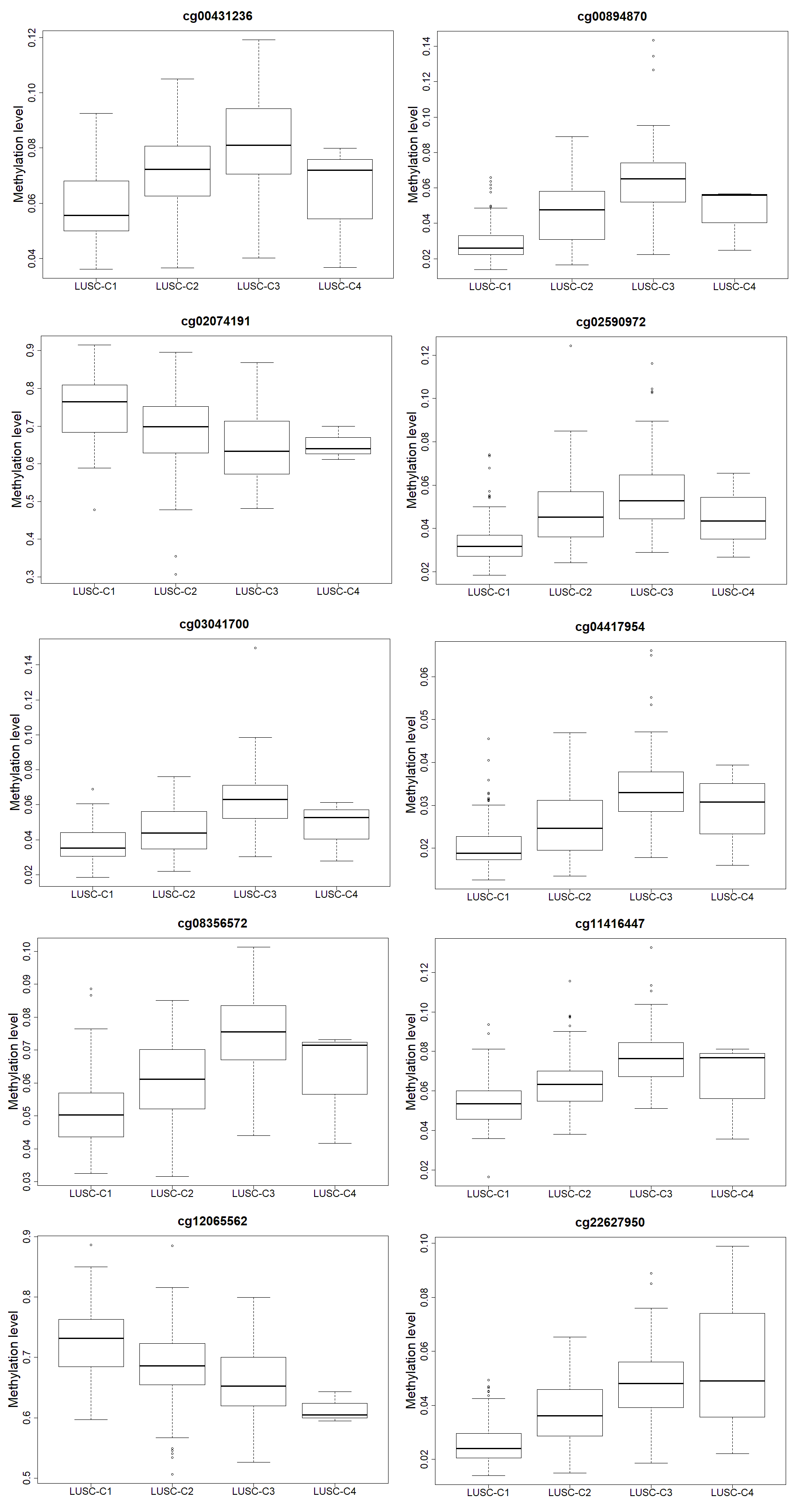


Supplementary fig. 4. Boxplots of 10 methylation site biomarkers for LUSC-C3.


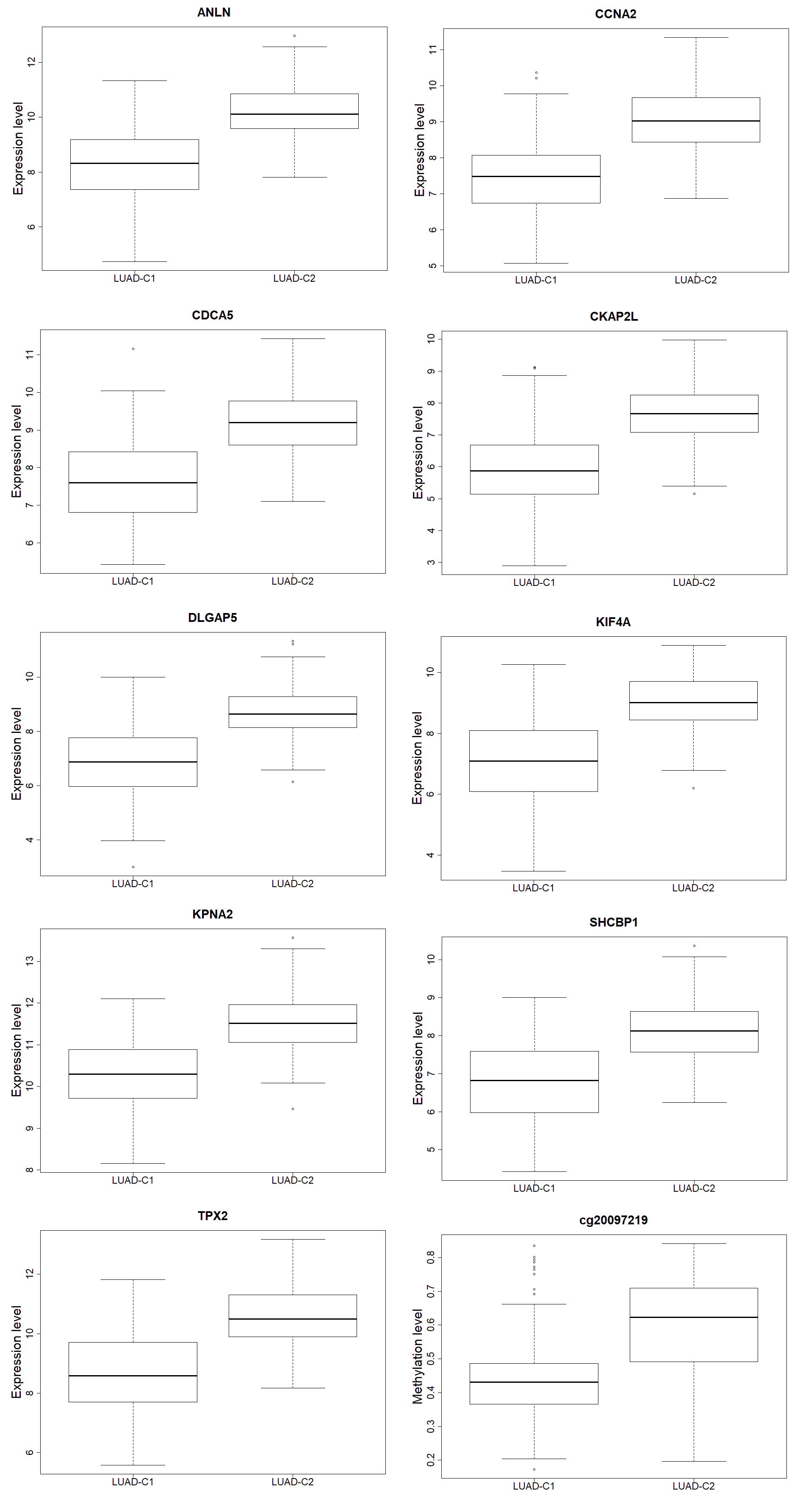


Supplementary fig. 5. Boxplots of 9 gene expression and 1 methylation site biomarkers for LUAD-C1.


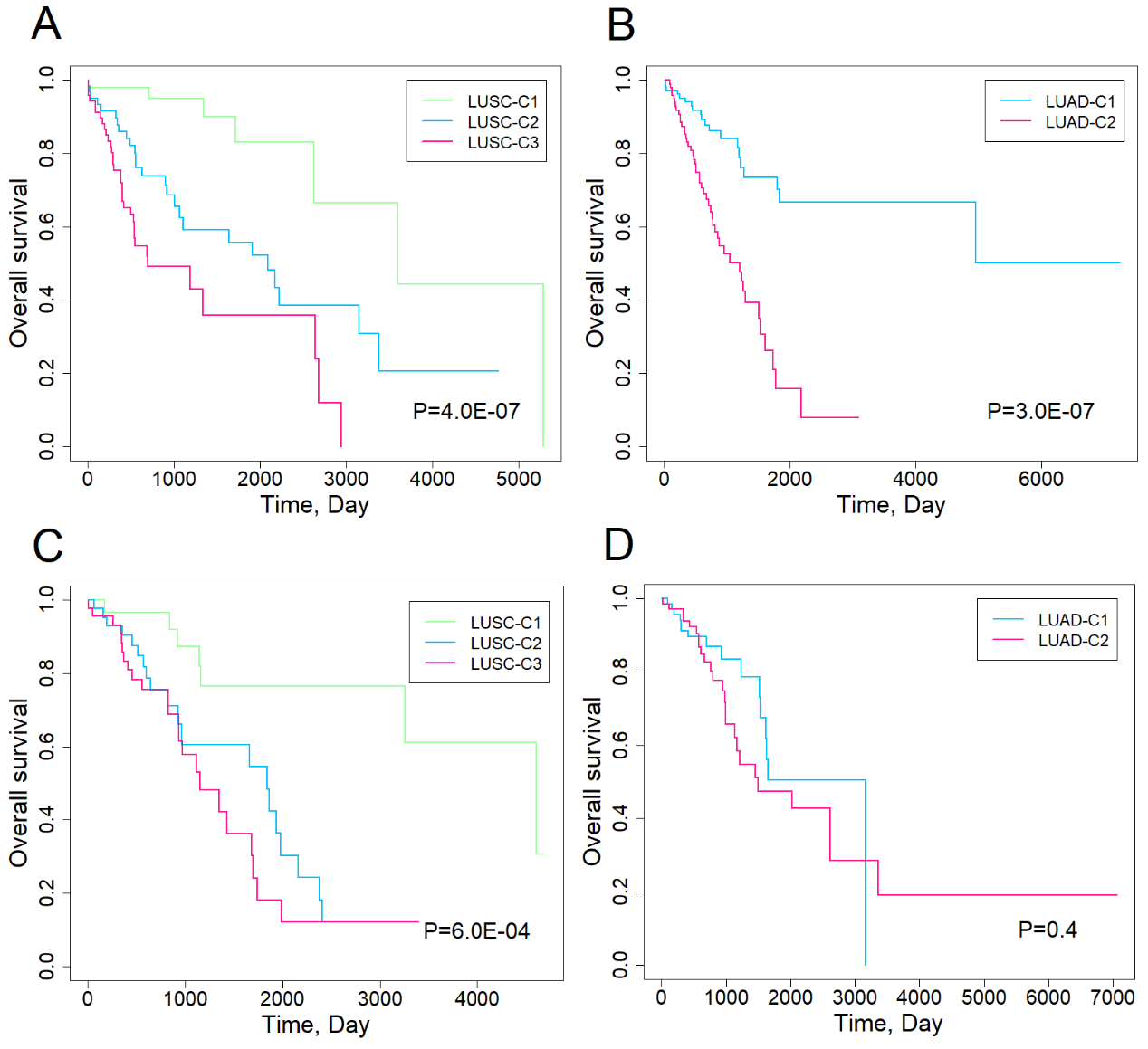


Supplementary fig. 6.The K-M survival curve for molecular subtypes and overall survival in different data set. (A) LUSC in training set. (B) LUAD in training set. (C) LUSC in test set. (D) LUAD in test set.

Supplementary table 1. Functional enrichment of differentially expressed genes and methylated genes related with overall survival for LUSC.

| **Category** | **Term** | **P value** |
| --- | --- | --- |
| **Differentially methylated genes** | |  |
| Biological process | GO:0007094~mitotic spindle assembly checkpoint | 2.09E-03 |
| Biological process | GO:0045141~meiotic telomere clustering | 5.69E-03 |
| Biological process | GO:0006351~transcription, DNA-templated | 7.07E-03 |
| Biological process | GO:1901796~regulation of signal transduction by p53 class mediator | 2.29E-02 |
| Biological process | GO:0071333~cellular response to glucose stimulus | 2.99E-02 |
| Biological process | GO:0007050~cell cycle arrest | 3.69E-02 |
| Biological process | GO:0045893~positive regulation of transcription, DNA-templated | 3.70E-02 |
| Biological process | GO:0071930~negative regulation of transcription involved in G1/S transition of mitotic cell cycle | 3.84E-02 |
| Cellular component | GO:0030054~cell junction | 1.19E-02 |
| Cellular component | GO:0017053~transcriptional repressor complex | 3.05E-02 |
| Cellular component | GO:0005874~microtubule | 3.98E-02 |
| Cellular component | GO:0005634~nucleus | 4.53E-02 |
| Molecular function | GO:0003677~DNA binding | 9.88E-04 |
| Molecular function | GO:0008270~zinc ion binding | 8.06E-03 |
| Molecular function | GO:0005539~glycosaminoglycan binding | 1.98E-02 |
| Molecular function | GO:0008134~transcription factor binding | 2.30E-02 |
| Molecular function | GO:0017137~Rab GTPase binding | 2.39E-02 |
| Molecular function | GO:0003924~GTPase activity | 2.44E-02 |
| Molecular function | GO:0005525~GTP binding | 4.53E-02 |
| **Differentially expressed genes** | |  |
| Cellular component | GO:0005764~lysosome | 1.17E-01 |
| Cellular component | GO:0031965~nuclear membrane | 1.19E-01 |
| Cellular component | GO:0070062~extracellular exosome | 1.91E-01 |

Supplementary table 2. Functional enrichment of differentially expressed genes and methylated genes related with overall survival for LUAD.

| Category | Term | P value |
| --- | --- | --- |
| **Differentially methylated genes** | |  |
| Biological process | GO:0033539~fatty acid beta-oxidation using acyl-CoA dehydrogenase | 2.95E-03 |
| Biological process | GO:0035556~intracellular signal transduction | 6.83E-03 |
| Biological process | GO:0071230~cellular response to amino acid stimulus | 7.22E-03 |
| Biological process | GO:0046777~protein autophosphorylation | 7.52E-03 |
| Biological process | GO:0043065~positive regulation of apoptotic process | 1.09E-02 |
| Biological process | GO:0010821~regulation of mitochondrion organization | 1.85E-02 |
| Biological process | GO:0045944~positive regulation of transcription from RNA polymerase II promoter | 1.90E-02 |
| Biological process | GO:0051301~cell division | 2.98E-02 |
| Biological process | GO:0022008~neurogenesis | 3.12E-02 |
| Biological process | GO:0006094~gluconeogenesis | 3.51E-02 |
| Biological process | GO:0043547~positive regulation of GTPase activity | 4.60E-02 |
| Cellular component | GO:0005829~cytosol | 1.86E-03 |
| Cellular component | GO:0005874~microtubule | 3.86E-03 |
| Cellular component | GO:0005737~cytoplasm | 1.14E-02 |
| Cellular component | GO:0005634~nucleus | 1.21E-02 |
| Cellular component | GO:0005819~spindle | 1.21E-02 |
| Cellular component | GO:0005764~lysosome | 2.67E-02 |
| Cellular component | GO:0043231~intracellular membrane-bounded organelle | 3.06E-02 |
| Cellular component | GO:0032587~ruffle membrane | 4.01E-02 |
| Cellular component | GO:0044232~organelle membrane contact site | 4.63E-02 |
| Molecular function | GO:0003995~acyl-CoA dehydrogenase activity | 2.73E-02 |
| Molecular function | GO:0005524~ATP binding | 2.94E-02 |
| Molecular function | GO:0008022~protein C-terminus binding | 2.99E-02 |
| Molecular function | GO:0008574~ATP-dependent microtubule motor activity, plus-end-directed | 3.06E-02 |
| Molecular function | GO:0003677~DNA binding | 3.43E-02 |
| Molecular function | GO:0001077~transcriptional activator activity, RNA polymerase II core promoter proximal region sequence-specific binding | 3.99E-02 |
| Molecular function | GO:0003777~microtubule motor activity | 4.16E-02 |
| KEGG pathway | hsa00480: Glutathione metabolism | 1.46E-02 |
| KEGG pathway | hsa04210: Apoptosis | 2.78E-02 |
| **Differentially expressed genes** | |  |
| Biological process | GO:0032147~activation of protein kinase activity | 2.11E-04 |
| Biological process | GO:0007067~mitotic nuclear division | 2.27E-04 |
| Biological process | GO:0086073~bundle of His cell-Purkinje myocyte adhesion involved in cell communication | 6.27E-04 |
| Biological process | GO:0000281~mitotic cytokinesis | 8.83E-04 |
| Biological process | GO:0098911~regulation of ventricular cardiac muscle cell action potential | 2.25E-03 |
| Biological process | GO:0051301~cell division | 8.20E-03 |
| Biological process | GO:0051726~regulation of cell cycle | 8.96E-03 |
| Biological process | GO:0000086~G2/M transition of mitotic cell cycle | 1.26E-02 |
| Biological process | GO:0070194~synaptonemal complex disassembly | 1.31E-02 |
| Biological process | GO:0010800~positive regulation of peptidyl-threonine phosphorylation | 1.34E-02 |
| Biological process | GO:0043065~positive regulation of apoptotic process | 1.41E-02 |
| Biological process | GO:0007018~microtubule-based movement | 1.61E-02 |
| Biological process | GO:0007160~cell-matrix adhesion | 2.13E-02 |
| Biological process | GO:0086091~regulation of heart rate by cardiac conduction | 2.20E-02 |
| Biological process | GO:0009409~response to cold | 2.32E-02 |
| Biological process | GO:0090307~mitotic spindle assembly | 2.32E-02 |
| Biological process | GO:0007080~mitotic metaphase plate congression | 2.44E-02 |
| Biological process | GO:0055088~lipid homeostasis | 2.69E-02 |
| Biological process | GO:0006555~methionine metabolic process | 3.87E-02 |
| Biological process | GO:0051988~regulation of attachment of spindle microtubules to kinetochore | 4.50E-02 |
| Biological process | GO:0050900~leukocyte migration | 4.61E-02 |
| Cellular component | GO:0005737~cytoplasm | 9.96E-06 |
| Cellular component | GO:0005829~cytosol | 1.60E-05 |
| Cellular component | GO:0030496~midbody | 1.68E-04 |
| Cellular component | GO:0014704~intercalated disc | 2.86E-03 |
| Cellular component | GO:0005874~microtubule | 3.59E-03 |
| Cellular component | GO:0005871~kinesin complex | 4.56E-03 |
| Cellular component | GO:0030057~desmosome | 9.95E-03 |
| Cellular component | GO:0009986~cell surface | 2.15E-02 |
| Cellular component | GO:0005654~nucleoplasm | 2.36E-02 |
| Cellular component | GO:0005634~nucleus | 3.01E-02 |
| Cellular component | GO:0005876~spindle microtubule | 3.14E-02 |
| Cellular component | GO:0000922~spindle pole | 3.17E-02 |
| Molecular function | GO:0008574~ATP-dependent microtubule motor activity, plus-end-directed | 1.37E-04 |
| Molecular function | GO:0019901~protein kinase binding | 4.54E-04 |
| Molecular function | GO:0086083~cell adhesive protein binding involved in bundle of His cell-Purkinje myocyte communication | 5.34E-04 |
| Molecular function | GO:0003777~microtubule motor activity | 1.40E-03 |
| Molecular function | GO:0005524~ATP binding | 1.43E-03 |
| Molecular function | GO:0004674~protein serine/threonine kinase activity | 7.78E-03 |
| Molecular function | GO:0043236~laminin binding | 9.91E-03 |
| Molecular function | GO:0042802~identical protein binding | 1.51E-02 |
| Molecular function | GO:0003824~catalytic activity | 2.72E-02 |
| Molecular function | GO:0005515~protein binding | 3.01E-02 |
| Molecular function | GO:0008017~microtubule binding | 3.74E-02 |
| KEGG pathway | hsa05412: Arrhythmogenic right ventricular cardiomyopathy (ARVC) | 9.86E-04 |
| KEGG pathway | hsa05222: Small cell lung cancer | 2.39E-03 |
| KEGG pathway | hsa05145: Toxoplasmosis | 6.03E-03 |
| KEGG pathway | hsa04512: ECM-receptor interaction | 2.01E-02 |
| KEGG pathway | hsa04914: Progesterone-mediated oocyte maturation | 2.01E-02 |
| KEGG pathway | hsa00480: Glutathione metabolism | 4.53E-02 |
| KEGG pathway | hsa04510: Focal adhesion | 4.79E-02 |

Supplementary table 3. The correlation of methylation sites and located genes.

|  | Methylation site | Gene name | Correlation |
| --- | --- | --- | --- |
| LUSC-C1 | cg00894870 | MRTO4; KIAA0090 | 0.036; 0.262 |
|  | cg03041700 | ATAD3B | -0.048 |
|  | cg04738309 | C5orf13 | 0.023 |
|  | cg08356572 | TRIM27 | 0.167 |
|  | cg09844983 | RPA2 | -0.028 |
|  | cg11416447 | DMTF1 | -0.165 |
|  | cg15987088 | GHSR | -0.101 |
|  | cg17152757 | GHSR | -0.145 |
|  | cg22627950 | TMED4 | 0.178 |
|  | cg24599434 | GHSR | -0.144 |
| LUSC-C3 | cg00431236 | ACP1; SH3YL1 | 0.091; -0.075 |
|  | cg00894870 | MRTO4; KIAA0090 | 0.036; 0.262 |
|  | cg02074191 | PCDHGA1; PCDHGA2; PCDHGA3; PCDHGA4; PCDHGA5; PCDHGA6; PCDHGA7; PCDHGA8; PCDHGB1; PCDHGB2; PCDHGB3; PCDHGB4; PCDHGB5 | 0.067; -0.223; -0.21; -0.104; -0.221; -0.135; -0.141; -0.129; 0.093; -0.055; -0.058; -0.15; -0.222 |
|  | cg02590972 | RPL37A | -0.006 |
|  | cg03041700 | ATAD3B | -0.048 |
|  | cg04417954 | CRTC3 | 0.175 |
|  | cg08356572 | TRIM27 | 0.167 |
|  | cg11416447 | DMTF1 | -0.165 |
|  | cg12065562 | PCDHB18 | -0.3 |
|  | cg22627950 | TMED4 | 0.178 |

Supplementary table 4. Functional enrichment of 9 biomarkers for LUAD-C1.

| Category | Term | Genes | P value |
| --- | --- | --- | --- |
| Biological process | GO:0007067~mitotic nuclear division | KIF4A, DLGAP5, TPX2, ANLN, CDCA5, CCNA2 | 2.29E-07 |
| Biological process | GO:0000280~nuclear division | KIF4A, DLGAP5, TPX2, ANLN, CDCA5, CCNA2 | 1.03E-06 |
| Biological process | GO:0048285~organelle fission | KIF4A, DLGAP5, TPX2, ANLN, CDCA5, CCNA2 | 1.41E-06 |
| Biological process | GO:1903047~mitotic cell cycle process | KIF4A, DLGAP5, TPX2, ANLN, CDCA5, CCNA2 | 8.28E-06 |
| Biological process | GO:0000278~mitotic cell cycle | KIF4A, DLGAP5, TPX2, ANLN, CDCA5, CCNA2 | 1.25E-05 |
| Biological process | GO:0007346~regulation of mitotic cell cycle | DLGAP5, TPX2, ANLN, CDCA5, CCNA2 | 2.44E-05 |
| Biological process | GO:0044772~mitotic cell cycle phase transition | DLGAP5, TPX2, ANLN, CDCA5, CCNA2 | 2.92E-05 |
| Biological process | GO:0044770~cell cycle phase transition | DLGAP5, TPX2, ANLN, CDCA5, CCNA2 | 3.73E-05 |
| Biological process | GO:0051301~cell division | KIF4A, TPX2, ANLN, CDCA5, CCNA2 | 4.53E-05 |
| Biological process | GO:0010564~regulation of cell cycle process | DLGAP5, TPX2, ANLN, CDCA5, CCNA2 | 5.19E-05 |
| Biological process | GO:0022402~cell cycle process | KIF4A, DLGAP5, TPX2, ANLN, CDCA5, CCNA2 | 6.52E-05 |
| Biological process | GO:0007049~cell cycle | KIF4A, DLGAP5, TPX2, ANLN, CDCA5, CCNA2 | 1.79E-04 |
| Biological process | GO:1901990~regulation of mitotic cell cycle phase transition | DLGAP5, ANLN, CDCA5, CCNA2 | 2.16E-04 |
| Biological process | GO:1901987~regulation of cell cycle phase transition | DLGAP5, ANLN, CDCA5, CCNA2 | 2.68E-04 |
| Biological process | GO:0051726~regulation of cell cycle | DLGAP5, TPX2, ANLN, CDCA5, CCNA2 | 3.56E-04 |
| Biological process | GO:0007088~regulation of mitotic nuclear division | DLGAP5, ANLN, CDCA5 | 1.43E-03 |
| Biological process | GO:0000070~mitotic sister chromatid segregation | KIF4A, DLGAP5, CDCA5 | 1.47E-03 |
| Biological process | GO:0051783~regulation of nuclear division | DLGAP5, ANLN, CDCA5 | 1.96E-03 |
| Biological process | GO:0000819~sister chromatid segregation | KIF4A, DLGAP5, CDCA5 | 3.62E-03 |
| Biological process | GO:0098813~nuclear chromosome segregation | KIF4A, DLGAP5, CDCA5 | 5.87E-03 |
| Biological process | GO:0007096~regulation of exit from mitosis | ANLN, CDCA5 | 6.71E-03 |
| Biological process | GO:0007059~chromosome segregation | KIF4A, DLGAP5, CDCA5 | 7.88E-03 |
| Biological process | GO:0000281~mitotic cytokinesis | KIF4A, ANLN | 1.38E-02 |
| Biological process | GO:0061640~cytoskeleton-dependent cytokinesis | KIF4A, ANLN | 1.71E-02 |
| Biological process | GO:0090307~mitotic spindle assembly | KIF4A, TPX2 | 1.84E-02 |
| Biological process | GO:1902850~microtubule cytoskeleton organization involved in mitosis | KIF4A, TPX2 | 1.88E-02 |
| Biological process | GO:0045840~positive regulation of mitotic nuclear division | DLGAP5, CDCA5 | 1.88E-02 |
| Biological process | GO:0051785~positive regulation of nuclear division | DLGAP5, CDCA5 | 2.33E-02 |
| Biological process | GO:1902589~single-organism organelle organization | KIF4A, DLGAP5, TPX2, CDCA5 | 2.35E-02 |
| Biological process | GO:1901992~positive regulation of mitotic cell cycle phase transition | DLGAP5, CDCA5 | 2.41E-02 |
| Biological process | GO:0007017~microtubule-based process | KIF4A, DLGAP5, TPX2 | 2.55E-02 |
| Biological process | GO:1901989~positive regulation of cell cycle phase transition | DLGAP5, CDCA5 | 2.70E-02 |
| Biological process | GO:0033045~regulation of sister chromatid segregation | DLGAP5, CDCA5 | 2.78E-02 |
| Biological process | GO:0070925~organelle assembly | KIF4A, TPX2, ANLN | 2.83E-02 |
| Biological process | GO:0051303~establishment of chromosome localization | DLGAP5, CDCA5 | 2.87E-02 |
| Biological process | GO:0050000~chromosome localization | DLGAP5, CDCA5 | 2.91E-02 |
| Biological process | GO:0008283~cell proliferation | DLGAP5, TPX2, CCNA2, SHCBP1 | 3.45E-02 |
| Biological process | GO:0051983~regulation of chromosome segregation | DLGAP5, CDCA5 | 3.48E-02 |
| Biological process | GO:0051225~spindle assembly | KIF4A, TPX2 | 3.72E-02 |
| Biological process | GO:0051649~establishment of localization in cell | KIF4A, DLGAP5, CDCA5, KPNA2 | 4.19E-02 |
| Biological process | GO:0007051~spindle organization | KIF4A, TPX2 | 4.53E-02 |
| Biological process | GO:0000910~cytokinesis | KIF4A, ANLN | 4.94E-02 |
| Cellular component | GO:0005819~spindle | KIF4A, CKAP2L, DLGAP5, TPX2, SHCBP1 | 1.22E-05 |
| Cellular component | GO:0015630~microtubule cytoskeleton | KIF4A, CKAP2L, DLGAP5, TPX2, SHCBP1 | 1.79E-03 |
| Cellular component | GO:0000922~spindle pole | CKAP2L, DLGAP5, TPX2 | 2.25E-03 |
| Cellular component | GO:0005654~nucleoplasm | KIF4A, TPX2, ANLN, CDCA5, KPNA2, CCNA2 | 1.19E-02 |
